# Complementary and Integrative Health Approaches and Opioid Prescriptions Among Older Veterans With Chronic Pain

**DOI:** 10.1093/geroni/igab046.3375

**Published:** 2021-12-17

**Authors:** Ling Han, Joseph Goulet, Melissa Skanderson, Doug Redd, Cynthia Brandt, Qing Zeng

**Affiliations:** 1 Yale School of Medicine, New Haven, Connecticut, United States; 2 VA Connecticut Healthcare System, West Haven, Connecticut, United States; 3 Washington DC VA Medical Center, Washington DC, District of Columbia, United States; 4 Yale School of Public Health, West Haven, Connecticut, United States; 5 George Washington University, Washington DC, District of Columbia, United States

## Abstract

Complementary and integrative health (CIH) approaches are recommended in national guidelines as viable options for managing chronic pain and de-prescribing opioids. We followed 1,993,455 Veterans with musculoskeletal disorders for two years who were not using opioids at study entry. CIH exposure was ascertained from primary care visits for acupuncture, massage and chiropractic care via natural language processing and structured data. Opioid prescriptions during the 2-year follow-up were abstracted from Veterans Health Administration (VHA) electronic pharmacy records. Propensity score (PS) was used to match CIH recipients with non-recipients with most comparable baseline characteristics. Overall, 140,902 (7.1%) Veterans received CIH, with a prevalence of 2.7% for Veterans aged ≥ 65y, comparing to 6.3% and 10.5% for those aged 50-64y and ≤ 49y, respectively. Among the 1:1 PS-matched sub-cohort (136,148 pairs), Cox proportional hazard model revealed that time to fill first opioid prescriptions was significantly longer for CIH recipients (mean: 587 days) than non-recipients (mean: 491 days), with adjusted Hazard Ratio of 0.48 (95% Confidence Interval (CI): 0.45-0.51) for Veterans ≥ 65y, 0.44 (95% CI: (95% CI: 0.43-0.45) for 50-64y and 0.47 (95% CI: 0.46-0.48) for age ≤ 49y group (p value for interaction, 0.003). Sensitivity analyses among full cohort or modeling total supply of first opioid prescriptions derived consistent results. These findings suggest potential benefit of CIH use in delaying and reducing opioids prescriptions for patients with chronic pain and may have implication for older Veterans ≥ 65y who have been found less likely to seek CIH therapies than their younger counterparts.

